# Evaluation of the coarse-grained OPEP force field for protein-protein docking

**DOI:** 10.1186/s13628-016-0029-y

**Published:** 2016-04-21

**Authors:** Philipp Kynast, Philippe Derreumaux, Birgit Strodel

**Affiliations:** Institute of Complex Systems: Structural Biochemistry (ICS-6), Forschungszentrum Jülich GmbH, Jülich, 52425 Germany; Laboratoire de Biochimie Théorique, UPR 9080 CNRS, Institut de Biologie Physico-Chimique, Paris, 75005 France; Institut Universitaire de France, 103 Boulevard Saint-Michel, Paris, 75005 France; University Paris Diderot, Sorbonne Paris Cité, Paris, 75205 France; Institute of Theoretical and Computational Chemistry, Heinrich Heine University Düsseldorf, Universitätsstr. 1, Düsseldorf, 40225 Germany

**Keywords:** Protein–protein docking, Coarse graining, Rescoring, Flexible docking

## Abstract

**Background:**

Knowing the binding site of protein–protein complexes helps understand their function and shows possible regulation sites. The ultimate goal of protein–protein docking is the prediction of the three-dimensional structure of a protein–protein complex. Docking itself only produces plausible candidate structures, which must be ranked using scoring functions to identify the structures that are most likely to occur in nature.

**Methods:**

In this work, we rescore rigid body protein–protein predictions using the optimized potential for efficient structure prediction (OPEP), which is a coarse-grained force field. Using a force field based on continuous functions rather than a grid-based scoring function allows the introduction of protein flexibility during the docking procedure. First, we produce protein–protein predictions using ZDOCK, and after energy minimization via OPEP we rank them using an OPEP-based soft rescoring function. We also train the rescoring function for different complex classes and demonstrate its improved performance for an independent dataset.

**Results:**

The trained rescoring function produces a better ranking than ZDOCK for more than 50 % of targets, rising to over 70 % when considering only enzyme/inhibitor complexes.

**Conclusions:**

This study demonstrates for the first time that energy functions derived from the coarse-grained OPEP force field can be employed to rescore predictions for protein–protein complexes.

## Background

One of the main goals of proteomic research is to understand the biological function of proteins. Many proteins generate their function not as monomers but as part of complexes. Thus knowledge about protein–protein interactions is fundamental and allows regulation of protein structure and function. The Protein Data Bank (PDB) [1] contains more than one hundred thousand protein structures. However, structures of protein–protein complexes are often difficult to determine experimentally. These complexes are usually very big, which is a problem for elucidating structure via nuclear magnetic resonance (NMR), and the interactions are often too transient to be captured by X-ray crystallography.

Protein-protein docking is an *in silico* method for predicting the structures of protein–protein complexes. One can predict possible binding sites in a complex based on the protein structures in their unbound state. The binding partners can be single proteins or smaller protein–protein complexes. To increase computing efficiency, the proteins are usually modelled as rigid bodies at the first six-dimensional (6D) global search stage. Most of these global search methods are based on the convolution of grids, where the surface of the binding partners are parametrized such that an overlap between the surfaces of the two binding partners becomes possible. The aim of this surface description is to implicitly account for conformational changes upon binding. The convolution of the grids is accelerated by fast Fourier transformation (FFT) [2–5]. In the simplest approach, the convolution produces possible docking positions based solely on the shape of the proteins. However, more sophisticated grid maps exist which take chemical and knowledge-based properties into account. For refining the initial predictions, various methods are commonly applied, for instance Monte Carlo (MC) simulations [6, 7], clustering [8, 9], or side-chain optimization using rotamer libraries [10]. As computation time is usually the limiting factor, an MC simulation should start from a conformation close to the binding site. A complete global search with this method in a reasonable computing time would be impossible.

The global search, which is performed via ZDOCK in this study [11], usually finds many similar solutions [4]. Therefore, it is common practice to cluster and rerank the docking predictions. Reranking classifies and distinguishes native or near-native solutions from non-native or wrong predictions [12, 13]. The number of predictions in a cluster can also be used for reranking [14]. The aim of both approaches is to narrow down the list of possible interaction sites, significantly decreasing computational cost and effort for further analysis of the remaining docking predictions.

To investigate protein–protein complexes produced by ZDOCK, docking approaches that allow for more protein flexibility than ZDOCK with low time expenditure are needed. A coarse-grained force field should be a good choice here. Various coarse-grained force fields have already been developed for the treatment of protein–protein complexes, including the calculation of thermodynamic and structural properties of multi-protein complexes with relatively low binding affinities [15]. Coarse-grained models are also used for molecular dynamics (MD) simulations of protein–protein association [16, 17], where the proteins are modelled using the MARTINI force field [18, 19] or with a Go-model approach [20]. In the latter approach [17], the electrostatic and hydrophobic interactions between proteins are modelled via a Coulomb potential with a distance dependent dielectric constant and the Miyzawa-Jernigan potential [21].

In the current study, we apply the coarse-grained ‘Optimized Potential for Efficient structure Prediction’ (OPEP) [22] to the protein–protein docking problem. A coarse-grained force field is used because of the reduced number of degrees of freedom, making it computationally more efficient than an all atom potential. Moreover, it is believed that a coarse-grained model will smooth the underlying free energy landscape, facilitating exploration of the corresponding phase space [23]. OPEP has already been successfully employed with different techniques, including MD and MC simulations. It was applied to RNA/DNA/protein systems to investigate the effect of crowding, to amyloid formation, and for protein 3D structure prediction. A recent overview of OPEP and its applications can be found in [22]. This work investigates OPEP’s applicability to protein–protein complexes. To test its performance for protein–protein docking, the first step is to investigate the discriminating power of OPEP to distinguish between correctly and wrongly docked complexes. We use global docking predictions produced by ZDOCK which we coarse grain and energy minimize using OPEP, followed by rescoring with an OPEP-based soft potential. Moreover, we enhance the performance of the rescoring function via an iterative learning procedure and test the resulting scoring function on a subset of the Dockground benchmark [24].

## Methods

**Fig. 1:**
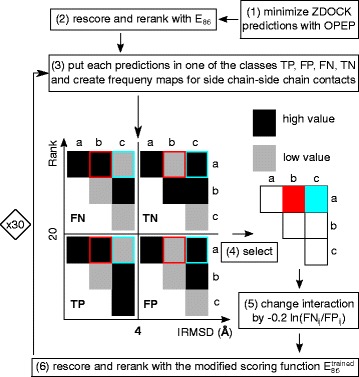
The training scheme for the side chain–side chain interactions. Every prediction is minimized (1) and rescored (2). Each prediction is classified as either TP, FP, FN, or TN (3). For each of these classes, an average contact map is created. Contact maps are shown for an artificial example containing only three residues. To train the potential, the side chain–side chain interaction a/b is selected because it is more frequent in TP and FN predictions than in FP and TN (4). The side chain–side chain interaction a/c, on the other hand, is selected because it is more frequent in FP and TN than in TP and FN predictions (4). The a/b interaction is strengthened by decreasing its energy, while the a/c interaction is disfavoured by increasing its energy (5). This leads to the new scoring function $E_{86}^{\text {trained}}$, with which the predictions are rescored. Steps (3) to (6) are iterated 30 times on the training dataset

We perform unbound docking, which starts from the binding partners in their native conformations. The methods applied for predicting and rescoring protein–protein complexes can be summarized via the following pipeline: For each of the 96 targets we produce 54,000 docking predictions with ZDOCK and retain the best 2000 of these complexes, as recommended by the ZDOCK developers. These predictions are energy minimized using the OPEP force field (step (1) in Fig. 1). For each prediction we perform 140 minimization steps in full Cartesian space with the limited-memory Broyden–Fletcher–Goldfarb–Shanno (LBFGS) minimizer [25], which leads to minimization times between 3.5 s for the target with PDB ID 1AY7 (185 amino acids) and 250 s for the target with PDB ID 2HMI (1413 amino acids) on a single CPU core. This amounts to an overall minimization time for the 2000 ZDOCK predictions per target of less than 24 h for 85 % of targets. Afterwards, the minimized predictions are reranked. For this, we replaced the side chain–side chain interaction potential of OPEP with a softer 8-6 Lennard-Jones-potential, while preserving the optimal distances and energies (step (2) in Fig. 1). At this stage, the OPEP potentials for salt bridges, interactions involving backbone atoms, and H-bonds are not changed. In a further step, we trained the parameters of side chain–side chain interactions, including salt bridge interactions with an iterative learning approach with the aim of further improving the performance of the OPEP-based rescoring function (steps (3)–(6) in Fig. 1). The resulting scoring function is tested on another dataset to independently prove its ability to distinguish between native and non-native complexes.

### The dataset

We use two different benchmarks to perform unbound docking. ZDOCK benchmark 4.0 is used as training dataset, while for further evaluation we use the Dockground benchmark 2.0. We used a subset of ZDOCK benchmark 4.0 [26]. We downloaded the docking predictions for 6° angular sampling from the ZDOCK website, which were obtained using ZDOCK 3.02 [27]. Ninety-six complexes were selected, including 39 enzyme/inhibitor, 19 antigen/antibody, and 38 other types of complexes. The latter will be called ‘other complexes’ for the remainder of this paper. One condition for selecting these complexes is that ZDOCK found at least one hit in the top 2000 predictions. A hit is defined as a prediction with an interface root mean square deviation (IRMSD) from the target of lower than 4 Å. Complexes that contain small molecules like ATP and GTP, for which OPEP is not parametrized, were not considered. The 1N2C complex could not be used, because it has more than 15,000 beads after coarse graining and the fixed file format for parametrization in OPEP currently only allows for up to 9999 beads.

The second dataset is a subset of the Dockground benchmark [24]. Here we follow the same selection criteria as for the ZDOCK benchmark. Furthermore, we remove complexes present in ZDOCK benchmark 4.0 in order to generate an independent and unbiased test set. The resulting test set contains 74 targets with 18 enzyme/inhibitor, 16 antigen/antibody, and 40 other complexes. As before, to generate complex predictions we applied ZDOCK with 6° sampling, using a local ZDOCK 3.02 installation and keeping the top 2000 predictions. As in the ZDOCK dataset, the docking for the antigen/antibody complexes was restricted to the complementarity determining regions (CDRs).

### ZDOCK

ZDOCK is an FFT-based rigid-body protein–protein docking algorithm. During the search procedure one protein is kept fixed, while the other is moved around it. The fixed protein is usually the larger of the two and is called the receptor, while the other protein is the ligand. ZDOCK generates grid-based representations from the full atom chains of receptor and ligand and after each ligand rotation the grids can be fast convoluted via FFT. The three rotational angles of the ligand are sampled with a 6° spacing, and the 3 translational degrees of freedom are sampled with a 1.2 Å spacing. For each set of rotational angles, only the best (based on ZDOCK score) translationally sampled prediction is retained [28]. This leads to 54,000 ZDOCK predictions, of which we consider the top 2000 for further refinement. To account for some flexibility in ZDOCK, a soft docking approach is used where the receptor has a 3.4 Å thick surface layer [3]. This allows for some overlap between receptor and ligand and accounts for possible movements during docking. However, it may also lead to atom clashes between receptor and ligand. The ZDOCK scoring function contains a shape-complementary term [29], a knowledge-based contact term for atoms and residues [11], and an electrostatic term [30].

### Missing residues and atoms

Some of the complex structures considered are missing certain residues in the receptor and/or ligand. Although this is no problem for a grid-based method like ZDOCK, it must be resolved for treatment with OPEP. Missing residues lead to gaps in the backbone chain and, if untreated, they would be considered overstretched bonds. In order to resolve this problem, polypeptides with missing residues are treated as separate chains. The distance between the terminal carbon and the terminal nitrogen of the gap is kept fixed via a harmonic potential with the equilibrium distance equal to the initial gap length and a force constant of 100 kcal/(mol·Å^2^).

### OPEP

As rescoring function we use the coarse-grained potential OPEP or variations of it. OPEP uses a six bead representation for every amino acid except proline and glycine. The amino nitrogen N, the C_*α*_, and the carbonyl carbon C’ atoms of the backbone are each modelled by one bead. In addition, the hydrogen H of the amino-group and carbonyl oxygen O are explicitly represented. Side chains are described by only one bead, except for proline where all heavy side chain atoms are modelled. The local energy terms in OPEP were developed based on the functional form of the Amber force field [31] and several rounds of minor adaptations to the side chain–side chain interactions have been conducted [22]. We use the latest version of OPEP, OPEPv5 [32], which for the first time includes an explicit potential for salt bridges that were parametrized with an iterative Boltzmann inversion method with parameters extracted from all atom MD simulations. A complete description of the OPEP potential can be found in the original OPEP publications [22, 31–33]. Here, we only present the nonbonded interactions, as they are used to rescore the protein–protein complexes. The nonbonded potential consists of four terms: (1) van der Waals interactions involving backbone atoms (*E*_VDW_), (2) hydrophobic and hydrophilic side chain–side chain interactions (*E*_SS_), (3) hydrogen bond (H-bond) interactions between backbone atoms (*E*_HB_), and (4) a potential for salt bridges (*E*_SB_). Interactions between side chains *E*_SS_ are modelled differently for attractive and repulsive interactions [34]:



where *r*_*ij*_ is the distance between interacting beads *i* and *j*, the equilibrium distance *σ*_*ij*_ is correlated with $r_{ij}^{0}$ via

(3) $$ \sigma_{ij} \approx 1.0729 r_{ij}^{0} - 0.3992,   $$

*ε*_*ij*_ is the interaction strength, and

(4) $$\begin{array}{@{}rcl@{}} G\left(r_{ij}^{0}\right) = \left[-0.7 \mathrm{e}^{\left(2 \left(r_{ij}^{0}-0.5\right)/5.0\right)} \left(r_{ij}^{0}-0.5 \right)\right]^{6} \end{array} $$

**Fig. 2:**
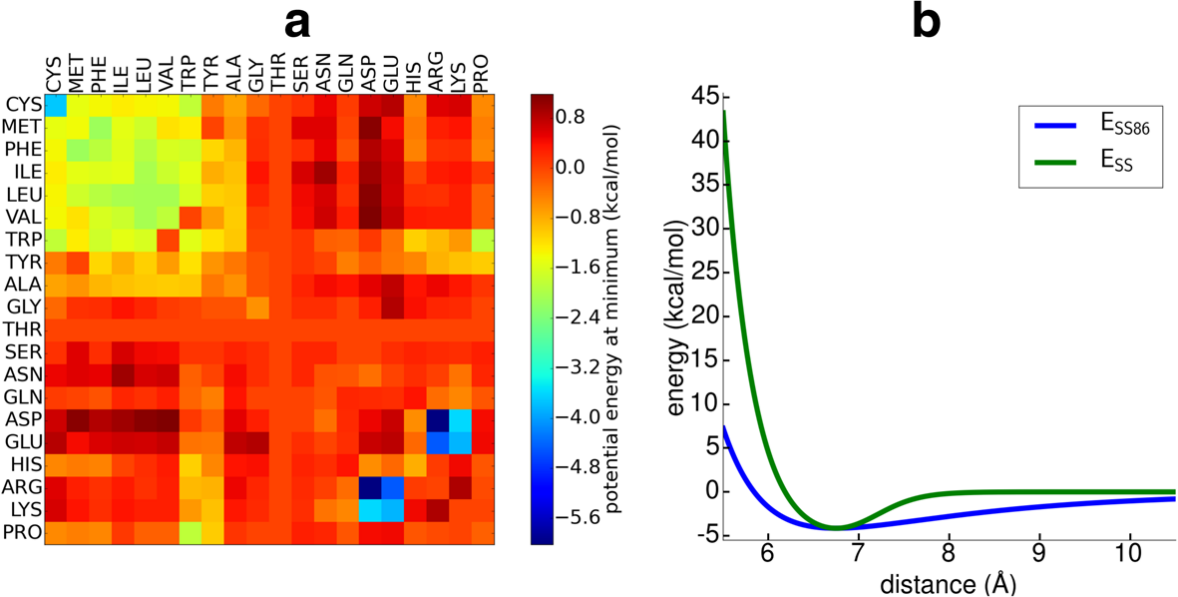
The OPEP force field. **a** The potential energy *E*_SS_+*E*_SB_ for side chain–side chain interactions is shown at the energy minimum, which is at *σ*_*ij*_ for *E*_SS_. For Arg/Asp and Arg/Glu, the average of the two minima for *E*_SB_ is shown. Repulsive interactions, corresponding to energies higher than zero, are also plotted at *σ*_*ij*_. **b** The OPEP potential *E*_SS_ is shown together with the soft function *E*_SS86_ for Phe/Phe

Figure 2a shows a matrix of the energies of the side chain–side chain interactions at the minimum distances *σ*_*ij*_. Equation (1) replaces the common 12-6 Lennard-Jones potential in order to limit *E*_SS_ at longer distances. Figure 2b shows an example of the form of the potential for the Phe/Phe interaction. For proline and glycine the center of interaction is the C_*α*_-atom, while for all other side chains the interaction center is a bead representing the center of mass of the side chain [33]. The potential *E*_SS_ is not used for salt bridges between side chains. Instead, salt bridges are modelled with a potential, *E*_SB_, derived from all atom MD simulations [32], where the distance dependent contact probability is translated to free energy profiles. These free energy profiles have one minimum for Arg/Asp and Arg/Glu pairs and two minima for Lys/Asp and Lys/Glu interactions. To describe backbone–backbone and backbone–side chain interactions, OPEP contains a van der Waals term, *E*_VDW_, which is modelled via a 12-6 Lennard-Jones potential. H-Bond interactions, *E*_HB_, are modelled between the backbone N-H and the backbone C’-O atoms. In addition, OPEP has special terms for stabilizing *α*-helices and *β*-sheets. The two-body term for H-bonds between residues in the same chain has different equilibrium distances for H-bonds less than five residues apart and for H-bonds further than four residues apart. For stabilizing *α*-helices, the intra-chain potentials also contain a 4-body H-bond term. Furthermore, 11 side chain–side chain interactions were identified to be more frequently found in (i, i + 3) and (i, i + 4) contacts in *α*-helices. Therefore, these side chain–side chain interactions with this particular separation were made more attractive [34].

### The scoring function

Before rescoring the predictions, we perform an energy minimization using OPEPv5 to relax the complexes after their transformation from the grid presentation to the coarse-grained model. We perform 140 minimization steps, as we found this to be the best compromise between computational efficiency and optimization result. We tested the effect of fewer and more minimization steps. Extending the minimization beyond 140 steps does not change the outcome of the rescoring result as for ∼90 % of the structures the energy only changes marginally at this point. Moreover, it happens especially for misdocked complexes that the energy minimum has not been reached within 140 minimization steps. However, there is no need to further optimize such misdocked decoys. Reducing the number of minimization steps below 140 bears the risk that also near-native structures have not been properly minimized yet, which would lead to a poor ranking for them. For the scoring function we found that it becomes more reliable if we introduce a softer potential, which allows for more overlap between the beads than the original OPEPv5 energy function. To obtain a softer scoring function we replace both the side chain–side chain interaction potential, *E*_SS_ from Eq. (1), and the 12-6 Lennard-Jones potential *E*_VDW_ with an 8-6 Lennard-Jones potential. This kind of soft potential is also used in the Attract force field that was developed for protein–protein docking [35]. We call the new potentials *E*_SS86_ and *E*_VDW86_, and the formula for *E*_SS86_ is given as:



Here, *σij*′=0.866*σ*_*ij*_ and *εij*′=9.481*E*_SS_(*σ*_*ij*_), with *σ*_*ij*_ given in Eq. (3). The values *σij*′ and *εij*′ are chosen such that the minimum energies at the equilibrium distances are identical for *E*_SS_ and *E*_SS86_. From Eq. (6), one can see that the repulsive-only potential is not modified. An example of the attractive *E*_SS86_ term is shown in Fig. 2b for the Phe/Phe side chain interaction. As the 8-6 potentials *E*_SS86_ and *E*_VDW86_ have broader wells than in OPEPv5, some overlap between beads is tolerated and, in addition, imperfectly fitted contacts are more strongly attractive at larger distances. The potentials for H-bonds and salt bridges were not modified, leading to our new scoring function, *E*_86_, with the modified potentials *E*_VDW86_ and *E*_SS86_:

(7) $$ E_{86} = E_{\text{VDW86}}+ E_{\text{SS86}} + E_{\text{HB}} + E_{\text{SB}},   $$

which calculates the binding energy between receptor and ligand for scoring purposes. It should be noted that each binding partner can consist of several proteins (chains). We consider all chains from one binding partner as a single protein. Hence, we only consider non-bonded energies between the two binding partners, e.g., between receptor and ligand.

### Interface RMSD

The interface RMSD (IRMSD) is defined as the RMSD between C_*α*_ interface atoms of the co-crystallized model and the prediction after superposition. Interface C_*α*_ atoms are all atoms within 10 Å distance of the binding partner in the co-crystallized complex [36]. For the superposition we use the corresponding function from Biopython [37].

### Definition of a hit

As is standard [38, 39], we define a hit as a docked conformation with an IRMSD lower than 4 Å.

### Performance evaluation

The performance is evaluated by ranking the predictions according to their (re)scoring energy in increasing order. From this list, the best ranked prediction with an IRMSD lower than 4 Å is reported. Furthermore, we calculate the success rate, which is a function of the number of predictions, *N*_pred_, that we consider from the sorted prediction list. This is averaged over the number of targets, *N*_target_, and is calculated according to following equation:

(8) $$ \text{success rate}(N_{\text{pred}}) = \frac{1}{N_{\text{target}}}\sum_{i=1}^{N_{\text{target}}} S_{i}(N_{\text{pred}}),   $$

where *S*_*i*_(*N*_pred_)=1 when the subset of *N*_pred_=1,2,…,2,000 predictions contains at least one hit, otherwise *S*_*i*_(*N*_pred_)=0. Thus, the success rate corresponds to the probability of finding the native complex among the *N*_pred_ first models based on the (re)scoring energy.

### Training the scoring function

After minimization, a residue-residue contact map between receptor and ligand is produced for each prediction. A contact is present if any of the beads of two residues are closer than 8 Å. Depending on the ranking with *E*_86_, one can classify the predictions for each complex into one of the four groups: true positive (TP), false negative (FN), false positive (FP), and true negative (TN). TPs have an IRMSD <4 Å and rank lower than or equal to 20, while the TN predictions have IRMSD ≥4 Å and a rank higher than 20. All other predictions are either FNs or FPs depending on whether their IRMSD is < or ≥ 4 Å and their ranking is > or ≤20. We only consider the first *N*=20 TPs or, if *N*<20 hits are found, we consider only those, because ideally one wants the correct predictions within the top hits. Twenty complexes is a small enough number for further processing by computationally more expensive approaches and visual inspection. We further limit the number of FNs and FPs to 20−*N* for training purposes. Thus, we do not consider FN and FP predictions if ≥20 hits are found for a target, as for such targets *E*_86_ already produces satisfying results. For each TP, FN, FP, and TN prediction considered, we calculate the frequency map for residue-residue contacts and average them over all targets for the enzyme/inhibitor, antigen/antibody, and other complexes. Next, we select residue-residue contacts where the frequency is higher in the maps for TP and FN than for the FP and TN maps. We assume these contacts need to be strengthened, so current FN predictions become TP without further favoring FP predictions. Therefore, we decrease the energy value *E*_SS86_ or *E*_SB_ for this contact. The other contacts, for which we modify the potential, are those where the frequency of TPs and FNs is lower than FPs and TNs. It appears these contacts are not important for the complex class in question and should thus be disfavored, with the aim of transforming a current FP prediction into a TN prediction. Therefore, we increase *E*_SS86_ or *E*_SB_ for such contacts. Figure 1 illustrates the training procedure.

The amount of change for the selected interaction between residues *i* and *j* is determined by the ratio between the corresponding FN _*ij*_ and FP _*ij*_ frequencies. A value greater than one means this interaction energy has to be decreased, while the opposite indicates this interaction energy has to be increased. We do this by changing the interaction potentials *E*_SS86_(*i*,*j*) and *E*_SB_(*i*,*j*) according to

(9) $$ E^{\text{trained}}_{\mathrm{X}}(i,j)=E^{\text{old}}_{\mathrm{X}}(i,j) - k \ln\left(\frac{\text{FN}_{ij}}{\text{FP}_{ij}}\right),   $$

where *E*_*X*_=*E*_SB_ or *E*_X_=*E*_SS86_ depending on the residue contact (*i*,*j*). For the parameter *k*, values between 0.1 and 0.6 were tested, and *k*=0.2 was found to be optimal. Equation (9) was iteratively applied. Thus, we had to determine when to stop the training for best parametrization and to avoid overfitting. To this end, we performed a 4-fold cross-validation on the enzyme/inhibitor training dataset, which gives us meaningful numbers for training and validation. This enzyme/inhibitor set contains 39 targets, of which 29 complexes were used for training, with the remaining 10 used for cross-validation. For these 10 targets, we measured the quality with $\sum _{i=1}^{10} \ln (\text {rank}(\text {target}_{i}))$, where rank() returns the rank of the best ranked hit. This function should decrease during training, while an increase is indicative of overfitting. We observe that overfitting becomes an issue after 30 iterations of Eq. (9). Therefore, we set the number of learning iterations to 30, yielding our new scoring function $E_{86}^{\text {trained}}$:

(10) $$ E_{86}^{\text{trained}}=E_{\text{VDW86}}+ E_{\text{SS86}}^{\text{trained}} + E_{\text{HB}} + E_{\text{SB}}^{\text{trained}}   $$

## Results

### Overall performance

**Fig. 3:**
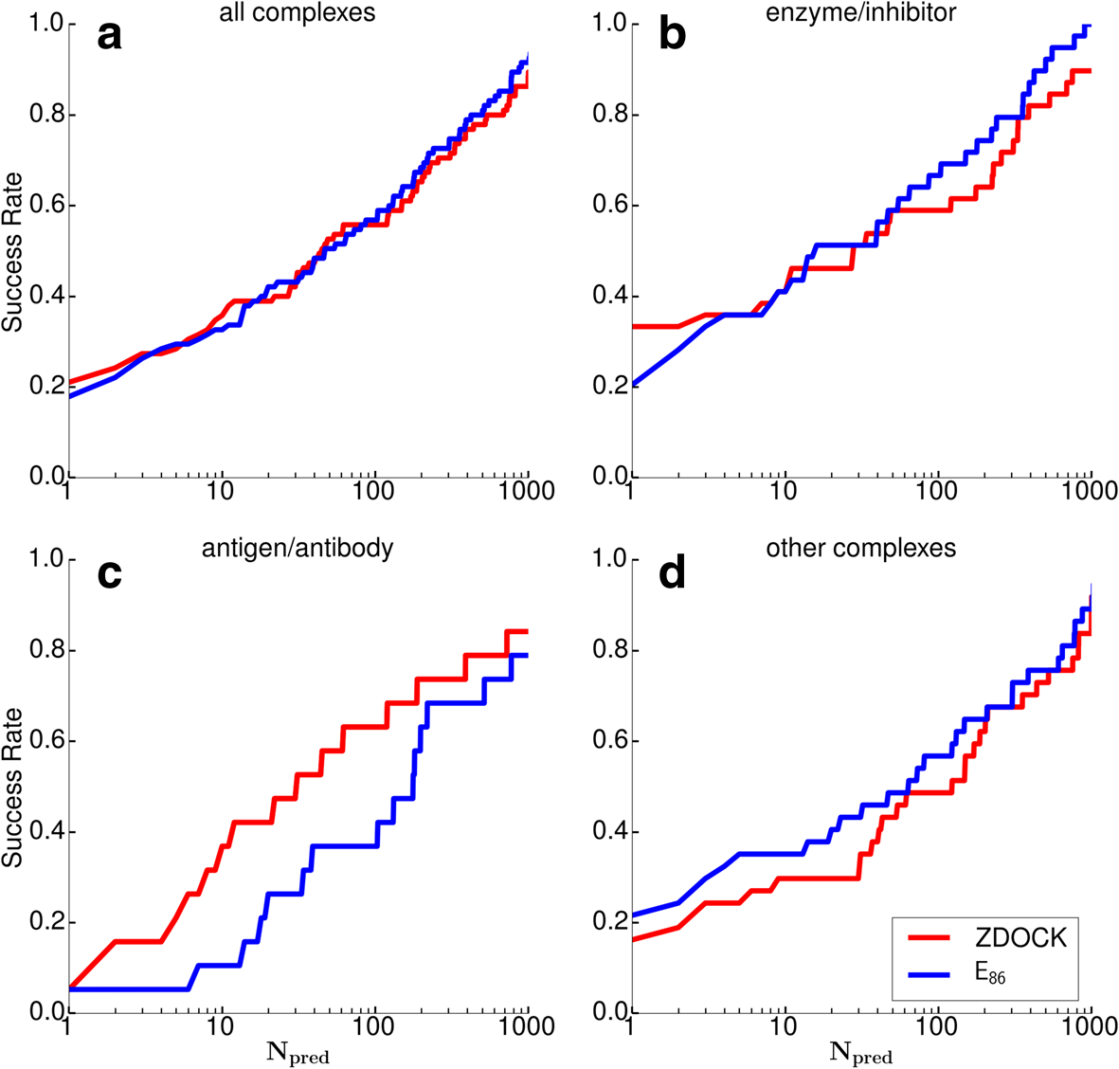
Results for the training dataset ZDOCK benchmark 4.0. The success rate is shown for (**a**) all complexes and separately for the three complex classes, (**b**) enzyme/inhibitor, (**c**) antigen/antibody and (**d**) other complexes using ZDOCK (*red*) and *E*_86_ (*blue*)

The ranks of the first hit using ZDOCK and after rescoring are shown in Table 1. The ZDOCK column gives the results for ZDOCK 3.02. The $E_{86}^{\text {initial}}$ column shows the rank after rescoring using Eq. (7) before energy minimization with the OPEP potential, while the *E*_86_ column reports the rank after minimization. Column five reports the rank of the first hit when using all intra- and interprotein contributions of the original OPEPv5 potential [32], while column six shows the rank of the first hit when the predictions are ranked by OPEPv5 energy when only the non-bonded energies between beads from the receptor and ligand are considered. These rescoring energies are denoted by *E*_OPEP_ and $E^{\text {int}}_{\text {OPEP}}$ in the following. Figure 3 represents the success rate as defined in Eq. (8) for the different complex classes. In general, ZDOCK and *E*_86_ perform better than *E*_OPEP_ and $E^{\text {int}}_{\text {OPEP}}$ and their performance is about equal if one considers the overall performance for all complex classes (Fig. 3a). However, there are differences between the three complex classes.

**Table 1:** Best rank for (re)scoring with ZDOCK, $E_{86}^{\text {initial}}$, *E*_86_, *E*_OPEP_, and $E_{\text {OPEP}}^{\text {int}}$ for complexes from the ZDOCK benchmark 4.0. $\varnothing $ indicates the average rank for the complex class in question

Complex	ZDOCK	$E_{86}^{\text {initial}}$	*E*_86_	*E*_OPEP_	$E_{\text {OPEP}}^{\text {int}}$
Enzyme/inhibitor					
1ACB	47	11	14	1	16
1AVX	1	128	3	27	31
1AY7	330	205	358	730	858
1BVN	1	39	1	98	1
1CGI	28	52	9	2	70
1CLV	1	2	1	1	1
1DFJ	1	102	1	14	4
1EAW	332	194	151	469	124
1EZU	121	919	11	946	559
1F34	176	428	2	1664	79
1FLE	1797	179	223	35	424
1GL1	49	116	8	107	34
1GXD	229	84	2	4	47
1HIA	389	88	901	1300	1392
1IJK	1296	924	355	70	5
1JIW	1350	504	553	989	851
1JTG	1	5	1	23	9
1MAH	1	80	87	9	27
1NW9	750	105	392	857	569
1N8O	11	11	14	9	12
1OC0	225	82	240	70	83
1OPH	28	150	422	903	822
2O8V	34	20	502	112	308
1OYV	3	34	1	2	381
1PPE	1	7	1	1	2
1R0R	533	896	40	151	856
1TMQ	7	228	3	295	1
1UDI	9	446	2	158	2
1YVB	11	86	47	220	67
2ABZ	689	670	772	619	1121
2B42	1	57	40	44	192
2J0T	1730	677	179	579	178
2MTA	1	122	104	13	43
2OUL	1	30	1	23	1
2SIC	1	145	1	3	5
3SGQ	309	158	16	596	260
2UUY	258	279	65	180	361
4CPA	1	1	4	1	20
7CEI	1	30	55	51	9
$\varnothing $	275.7	212.7	143.1	291.7	251.9
Antigen/antibody					
1AHW	1387	542	1087	1161	1563
1BJ1	1	20	132	89	230
1BVK	10	143	356	6	283
1DQJ [a]	1671	150	–	–	–
1E6J	8	8	39	21	10
1FSK	5	5	181	135	373
1I9R	31	39	177	23	109
1IQD	2	41	1	4	1
1JPS	1261	221	14	1635	1930
1KXQ	6	192	34	532	9
1K4C	120	201	1077	14	166
1MLC	188	1194	104	395	314
1NCA	389	331	774	1091	1340
1VFB	45	163	198	33	586
2FD6	2	82	20	16	268
2HMI	62	178	516	4	766
2I25	12	2	7	45	39
2JEL	22	85	18	49	139
2VIS	725	557	1538	625	1095
$\varnothing $	313.0	246.1	435.4	414.6	485.3
Other complexes					
1AKJ	754	47	1823	787	1887
1B6C	1	13	1	1	1
1BUH	62	25	304	10	2
1DE4	4	472	38	39	52
1F51	9	22	32	166	176
1FC2	1286	1672	1654	896	224
1FFW	41	47	47	111	433
1GLA	210	326	1013	329	1296
1GPW	3	2	1	111	997
1H9D	203	53	386	297	345
1HE1	821	1837	871	1907	1702
1I2M	354	828	3	482	262
1JK9	998	735	305	891	552
1JZD	31	141	208	3	92
1K74	1	26	1	110	717
1ML0	1	12	2	2	3
1RV6	1	188	4	1	2
1S1Q	1696	925	780	1635	932
1SYX	149	345	123	24	781
1T6B	439	224	14	130	30
1US7	150	107	772	283	341
1WDW	1	3	23	12	326
1XD3	3	25	1	1	1
1XU1	37	69	5	6	1
1Z5Y	31	1	1	1	1
1ZHI	187	43	608	156	471
2AJF	1115	318	1029	195	494
2A5T	824	421	148	573	1149
2AYO	6	112	3	700	902
2CFH	1	18	1	3	3
2HLE	54	29	20	52	74
2HRK	2	76	64	30	5
2IDO	171	314	73	577	11
2NZ8	1002	281	81	793	243
2VDB	43	36	1	13	309
2Z0E	123	181	187	684	1346
3BP8	998	375	131	538	242
3D5S	524	29	1	34	246
$\varnothing $	324.6	273.1	283.1	331.1	438.2

[a] The rank is set to 2000 for calculating the average

#### Enzyme/inhibitor

For enzyme/inhibitor complexes, *E*_86_ finds equal or more hits if more than four predictions are considered, i.e., *N*_pred_≥5 (Fig. 3b). When considering more than 50 predictions, *E*_86_ becomes substantially better than ZDOCK. Table 1 shows that we can improve or maintain the rank using *E*_86_ for 25 out of 39 enzyme/inhibitor targets. For 1AVX, the rank is only slightly worse, increasing from 1 with ZDOCK to 3 with *E*_86_. Comparing the performance of *E*_86_ to $E^{\text {int}}_{\text {OPEP}}$, it becomes evident that the 140 minimization steps are not always sufficient to put every side chain in the minimum of the well, because the rank with $E^{\text {int}}_{\text {OPEP}}$ is considerably higher than for *E*_86_. Thus rescoring with the softer potential is necessary. When using *E*_OPEP_ for ranking, the ranks of only 16 targets are kept or improved. The average rank shows that *E*_86_ is generally better than ZDOCK, while $E^{\text {int}}_{\text {OPEP}}$ produces a similar ranking to ZDOCK, and *E*_OPEP_ performs worst.

#### Antigen/antibody

For antigen/antibody complexes, rescoring with *E*_86_ was least successful. For *N*_pred_≲500, the success rate of *E*_86_ is clearly smaller than for ZDOCK (Fig. 3c). Out of 19 antigen/antibody complexes, *E*_86_ improves the rank for only six targets and worsens it for the other 13. Using *E*_OPEP_ only improves the ranking of six complexes, while the rank of only one complex can be improved using $E^{\text {int}}_{\text {OPEP}}$. The average rank shows that ZDOCK performs considerably better than any of the OPEP-based rescoring approaches. However, it should be noted that ZDOCK is not a perfect scoring function either for antigen/antibody complexes, as revealed by comparing the average ZDOCK ranks with enzyme/inhibitor complexes.

#### Other complexes

For other complexes, the success rate is always higher for rescoring with *E*_86_ than scoring with ZDOCK, independent of the number of predictions considered (Fig. 3d). The *E*_86_ score improves or maintains the rank of 21 complexes and worsens it for the other 17; however, for 1ML0 the rank only changes from 1 to 2 and 1RV6 from 1 to 4. While *E*_OPEP_ improves the rank of 20 targets and worsens the rank of 18 targets, the improvements mostly occur for higher ranks, and only four predictions have rank 1, compared with eight for *E*_86_. $E^{\text {int}}_{\text {OPEP}}$ can improve the rank of only 15 targets; it worsens the rank of the other 23. On average, for other complexes rescoring with *E*_86_ performs best, $E^{\text {int}}_{\text {OPEP}}$ is least suited for this task, and *E*_OPEP_ predicts a similar ranking as ZDOCK. From the strikingly different performance of *E*_86_ and $E^{\text {int}}_{\text {OPEP}}$ it seems that optimal shape complementarity implying favourable residue-residue interactions are very important for protein binding in this complex category.

### Structural changes upon energy minimization

We tested whether the structures of the complexes are affected as a result of energy minimization with the OPEP potential. To this end, the secondary structures of the complexes are determined before and after their energy minimization using STRIDE [40]. Since we use crystal structures of the unbound receptor and ligand as input, all 2000 ZDOCK predictions per target have the same secondary structures before minimization, while the secondary structures can change during minimization with the OPEP potential. However, we find that the changes in secondary structure are generally small (<5 %). Especially the near-native structures with IRMSD <3 Å are least affected by energy minimization, indicating that the correct binding helps stabilize the complex structure. However, the overall changes of secondary structure are small and do not follow a pattern, which prevents us from generalizing a dependency between IRMSD and secondary structure.

**Fig. 4:**
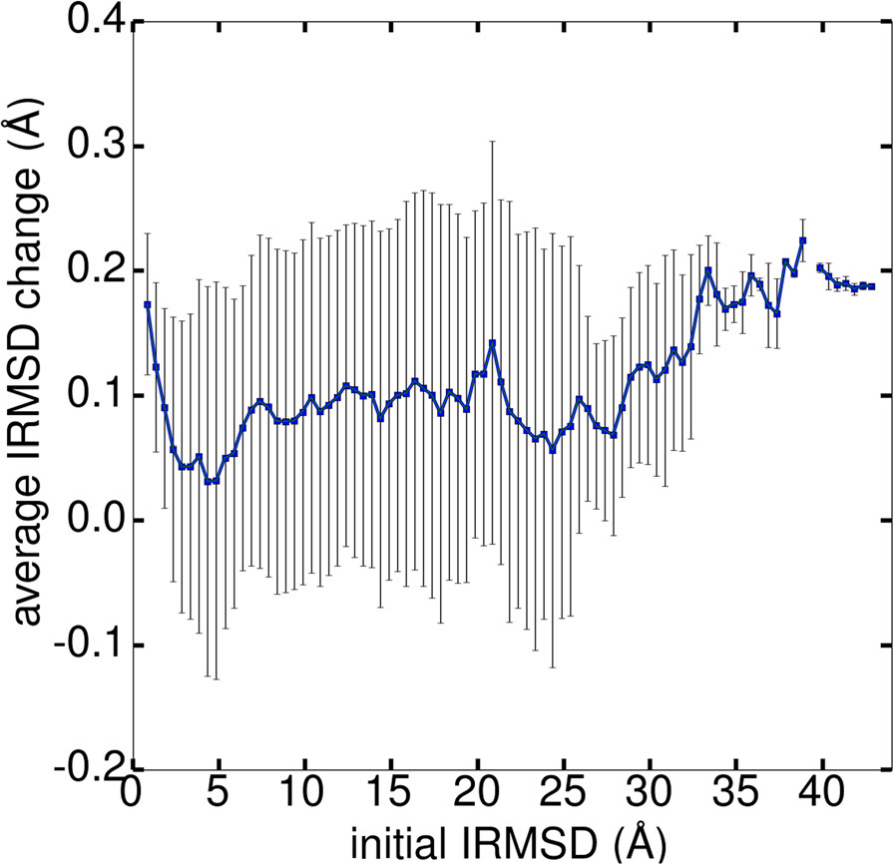
The average change in IRMSD as a result of energy minimization of the ZDOCK predictions using OPEP. Averages, calculated over all targets and complex classes, are shown in blue together with one standard deviation

We further tested if the IRMSD is affected by minimization with OPEP and found it changes only slightly. A plot showing the average change of IRMSD as a function of the initial IRMSD as obtained from ZDOCK can be seen in Fig. 4. For most predictions, the IRMSD slightly increases due to minimization with the average IRMSD change fluctuating aroud 0.1 Å. For some of the complexes, the IRMSD also decreases: for 4.3 % of the predictions with IRMSD <4 Å before minimization, which increases to 8.7 % if one considers all predictions. The preferred IRMSD increase for near-native predictions is likely to be an effect of the tight packing at the binding site, which leads to more bead clashes after transformation from the grid to the coarse-grained representation, causing the atoms or beads to reorient during minimization. Nonetheless, the structures stay close to the conformations predicted by ZDOCK, as Fig. 4 testifies. Only for severely misdocked complexes (IRMSD $\gtrsim 35$ Å) the IRMSD change increases to around 0.2 Å.

Comparison of columns three and four of Table 1 reveals the effect of minimizing the energy before rescoring with *E*_86_. Column three reports the best rank without energy minimization, which we denote as $E_{86}^{\text {initial}}$. For the comparison we concentrate on the complexes for which either *E*_86_ or $E_{86}^{\text {initial}}$, or both, predict a best rank ≤10 as in the Critical Assessment of PRedicted Interactions (CAPRI) experiment [41] one can only upload 10 predictions per target. Thus, the aim is to score the decoys closest to the native structure in the top 10. For enzyme/inhibitor complexes, energy minimization is most successful as *E*_86_ identifies for more than 38 % a hit in the top 10 predictions (see success rate for *N*_pred_=10 in Fig. 3b). For only four of these 15 complexes (namely 1CLV, 1JTG, 1PPE and 4CPA) also $E_{86}^{\text {initial}}$ predicts best ranks in the top 10, while it does not occur for enzyme/inhibitor complexes that $E_{86}^{\text {initial}}$ finds a hit in the top 10, which is lost upon energy minimization. In two cases (1F34 and 1UDI) energy minimization improves the rank by more than 400 places, leading to second places in the rank list. A similar picture emerges for other complexes, for which for more than 34 % of the complexes a best rank in the top 10 is found with *E*_86_ (see success rate for *N*_pred_=10 in Fig. 3d). With $E_{86}^{\text {initial}}$, on the other hand, for only three complexes a top-10 rank is achieved. For one of these three (1WDW) the rank increases from 3 to 23 upon energy minimization, while the other two are also top-10 ranked with *E*_86_. Only for antigen/antibody complexes preceding energy minimization of the complexes offers no advantage over direct application of the rescoring function. *E*_86_ and $E_{86}^{\text {initial}}$ find for 2 and 3, respectively, of the 19 complexes a hit in the top-10 rank list. For two complexes (1E6J and 1FSK) the top-10 rank is lost after energy minimization, while for 1IQD the best rank climbed 40 places and is ranked first with *E*_86_. However, it should be noted that the average rank for $E_{86}^{\text {initial}}$ is considerably lower than for both ZDOCK and *E*_86_. Thus, energy minimization of antigen/antibody complexes is not absolutely necessary. Though apart from saving us computing time, omitting this step would also not (considerably) increase our chances of identifying the right prediction as the increase of the average rank for *E*_86_ originates mainly from further deterioration of the already high ranks obtained with $E_{86}^{\text {initial}}$ (e.g., complexes 1AHW, 1K4C and 2VIS). More crucial would be a general improvement of the *E*_86_ scoring function for its application to antigen/antibody complexes.

### Energy contributions to the protein-protein interactions

**Fig. 5:**
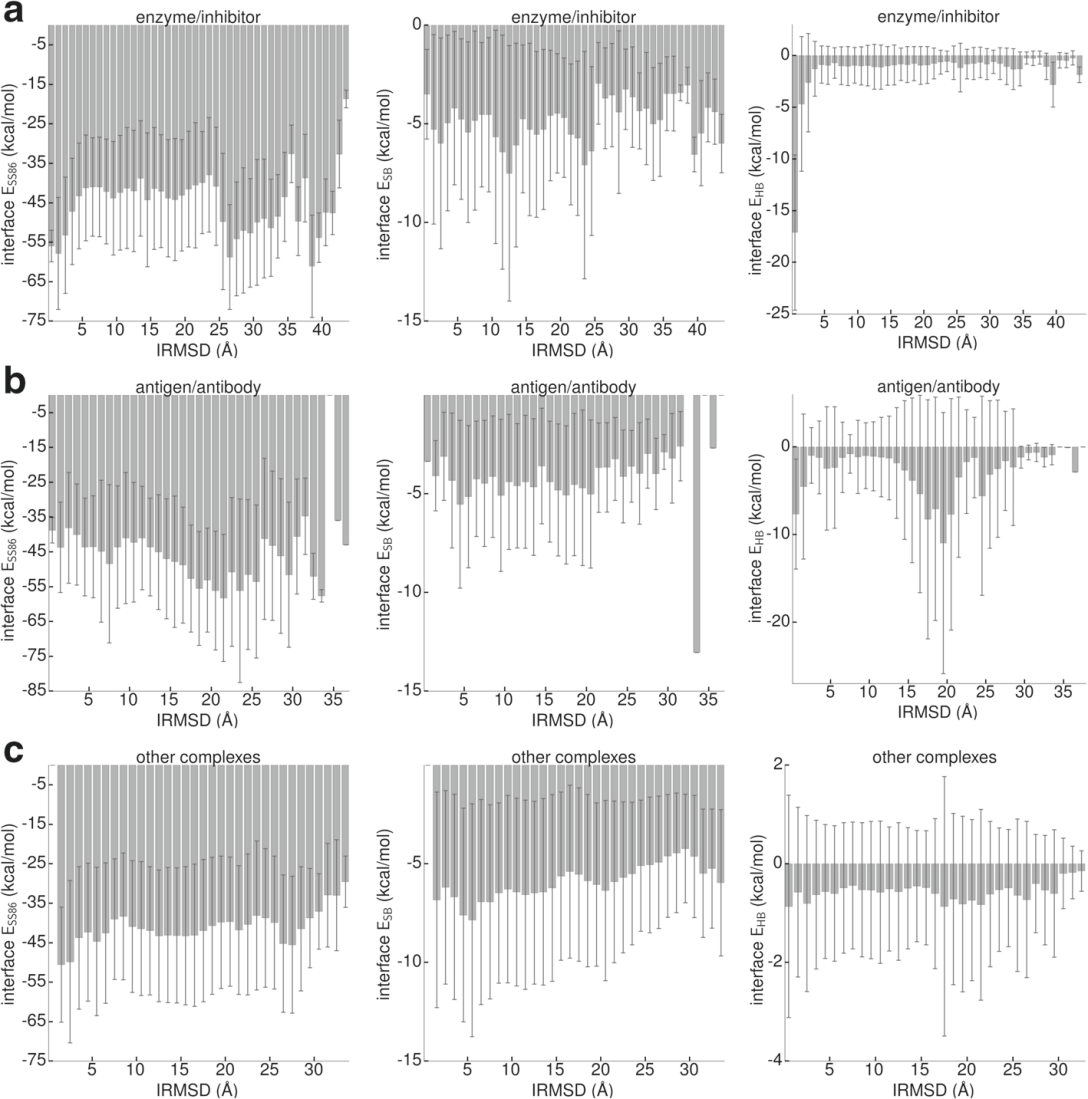
The different contributions of the rescoring function *E*_86_ for the different complex classes, (**a**) enzyme/inhibitor, (**b**) antigen/antibody and (**c**) other complexes as a function of the IRMSD: (*left*) *E*_SS86_, (*middle*) *E*_SB_, (*right*) *E*_HB_. Averages, calculated over all targets and predictions for each target belonging to one of the three complex classes, are shown together with one standard deviation

Figure 5 shows the different contributions to the *E*_86_ energy for predictions sorted by their IRMSD using a bin size of 1 Å. We show the averaged values of *E*_SS86_, *E*_SB_, and *E*_HB_ for the three complex classes. For the enzyme/inhibitor complexes, a minimum in *E*_SS86_ is present for predictions up to 5 Å. However, for IRMSD values above 25 Å *E*_SS86_ becomes small again, in some cases even smaller than for the hits. This is more than counterbalanced by the H-bond energy, as only near-native hits have more and better oriented H-bonds, leading to *E*_HB_ values more than 10 kcal/mol smaller than for all other predictions. Salt bridges seem to be of minor importance for the protein binding in enzyme/inhibitor complexes, as there is no correlation between the *E*_SB_ values and the IRMSD, and the contribution of *E*_SB_ to *E*_86_ is generally small, with all values fluctuating around −5 kcal/mol. Thus, the sum of *E*_SS86_ and *E*_HB_ is mainly responsible for distinguishing between correct and incorrect complex predictions. This partly agrees with previous findings that protease-inhibitor complexes interact predominantly through main chain–main chain interactions [42], which are represented by H-bonds in the *E*_86_ function.

For antigen/antibody complexes, none of the three energy contributions clearly decreases with decreasing IRMSD. Instead, both *E*_SS86_ and *E*_HB_ adopt their smallest values for IRMSD ≈20 Å, which explains why *E*_86_ does not perform well for this complex class. Compared to enzyme/inhibitor complexes, backbone H-bonds are less important for the native complex. This agrees with the previous observation that antigen and antibody complexes predominantly bind through side chain–side chain or side chain–main chain interactions [42], which are represented by other contributions from *E*_86_ but not by *E*_HB_. For antigen/antibody complexes, the formation of salt bridges is also of minor importance. There is only one exception, at IRMSD ≈34 Å, where with *E*_SB_≈−13 kcal/mol the smallest salt bridge energy is observed, also taking the other two complex classes into account.

The hits for other complexes are stabilized by side chain–side chain interactions, as the lowest values for *E*_SS86_ are found for the complexes with IRMSD <4 Å. H-bonds seem to be of minor importance for binding receptor and ligand in this complex category, as all *E*_HB_ values are >−1 kcal/mol, an order of magnitude higher than those in enzyme/inhibitor and antigen/antibody complexes. On the other hand, other complexes are the only ones where salt bridges contribute to stabilizing the complexes, as for IRMSD >5 Å, *E*_SB_ increases. This trend only breaks for IRMSD ≤5 Å as *E*_SB_ does not further decrease for the near-native predictions. This means that either *E*_SS86_ dominates these binding modes or the *E*_86_ potential can be further improved in this range.

### Improving the rescoring function

**Fig. 6:**
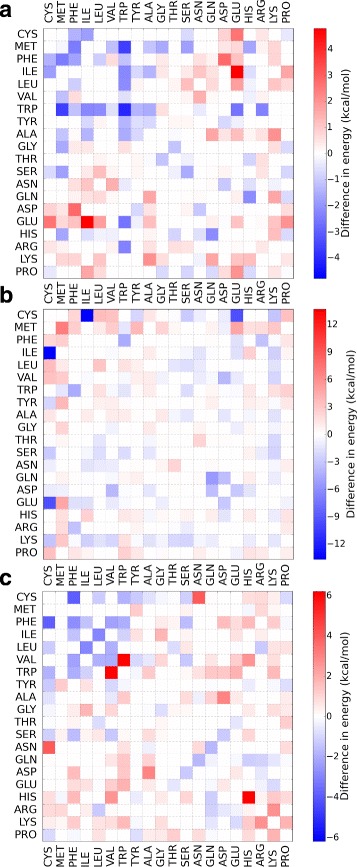
The change in minimum energy for side chain–side chain contacts as a result of training the scoring function *E*_86_ for (**a**) enzyme/inhibitor, (**b**) antigen/antibody, and (**c**) other complexes. *Blue* values mean the interaction became more attractive, *red* means the interaction became more repulsive. Note the different energy scales for the protein classes

Next we tested if the performance of *E*_86_ can be enhanced by training it according to Eq. (9), yielding the new rescoring function $E_{86}^{\text {trained}}$ defined in Eq. (10). As the energy analysis revealed that complex formation in the three categories is driven by different interactions, we decided to optimize *E*_86_ separately for enzyme/inhibitor, antigen/antibody, and other complexes. The resulting $E_{86}^{\text {trained}}$ leads to new energies at the optimal distances between the side chains at the binding sites, which can be presented as a matrix. Subtracting the new energy matrix from the original potential energy matrix shown in Fig. 2a gives a matrix for each complex category that represents the change in interaction energies. These matrices are shown in Fig. 6.

#### Enzyme/inhibitor

With few exceptions, the change in interaction energy follows the hydrophobicity of the amino acids. This confirms the findings from Fig. 5 that the enzyme/inhibitor complexes are stabilized by the interactions modeled by *E*_SS86_. The amino acids Phe to Ala are the hydrophobic amino acids, and interactions between them got stronger, except for Phe/Val and Phe/Ala. Most interactions involving polar amino acids do not change much, while some of the interactions involving charged residues become more repulsive. Previous studies also found that enzyme-inhibitor complexes contribute more hydrophobic interactions at the expense of polar contributions [42]. The most pronounced changes occur for Trp/Trp, Met/Trp, Glu/Ile, and Asp/Phe. The increased stability for Trp/Trp agrees with the Ravikant-Elber matrix [43], which was derived as the most likely interaction from a statistical analysis of protein–protein complexes. The Met/Trp interaction is also favoured by the Ravikant-Elber matrix. Both interactions were already attractive in the *E*_86_ rescoring function, but become even stronger as a result of the optimization procedure. Glu/Ile and Asp/Phe, on the other hand, were repulsive in *E*_86_ and become more so. Glu/Ile is also slightly repulsive in the Ravikant-Elber matrix, while Asp/Phe is slightly attractive. However, the Ravikant-Elber matrix includes protein–protein interactions independent of complex class, while our current finding only applies to enzyme/inhibitor complexes.

#### Antigen/antibody

Figure 6b shows the change in interaction energies for antigen/antibody complexes. Surprisingly, two interactions involving cysteine, namely Cys/Ile and Cys/Glu, considerably increase in strength. This probably results from the presence of a Cys residue just before the start of most of the CDRs [44], which is thus in contact with the antigen. The interaction between Met residues becomes the most repulsive. Before training it was slightly attractive. This change in energy is difficult to rationalize; many of the other changes are correlated to the frequency of residues at the antigen–antibody interface. At the paratope of the antibody, the residues that contribute most to the binding are Tyr, Trp, Asp, Glu, Asn, Ser, Thr, and Gly, while at the antigen epitope these are Arg, Lys, Asp, Tyr, Glu, Asn, Ser, Thr, and Gly [42]. Many of the interactions involving these residues become more attractive, while the remaining interactions do not change much in strength. This shows that our training scheme can strengthen interactions which have been previously shown to play an important role in antigen–antibody binding [42, 45].

#### Other complexes

The difference map for the other complexes can be seen in Fig. 6c. As with enzyme/inhibitor complexes, most hydrophobic interactions become more attractive. The exception is Trp/Val, for which the interaction became more repulsive. Previously, this interaction was only slightly repulsive. Almost all of the polar/hydrophobic interactions become more repulsive. Interestingly, the His/His interaction becomes considerably more repulsive, which corresponds well with repulsion of equal charges when His is positively charged. Before training, this interaction was attractive. The repulsion between the equally charged residues Glu/Asp and Arg/Lys also increased, but these were already repulsive before optimization. The salt bridges, with the exception of Lys/Asp, got stronger. Overall, this shows that electrostatics interactions play a more important role here than in the enzyme/inhibitor complexes. It also confirms the trend from Fig. 5, which revealed a general decrease (apart from a few exceptions) for *E*_SS86_ and *E*_SB_ with decreasing IRMSD.

### Test on a new dataset

To test the optimized rescoring function, we use the protein–protein complexes from the Dockground test set [24], removing all complexes which are also in the ZDOCK Benchmark 4.0 and were already used for training. The remaining 74 complexes are listed in Table 2. As before, we perform unbound docking with ZDOCK producing 2000 predictions for each target. However, ZDOCK is not able to produce a hit for all targets in the top 2000 predictions. In particular, ZDOCK is not very successful for other complexes, generating hits for only 19 out of 40 of these complexes. However, it is successful for 15 out of 18 enzyme/inhibitor complexes and 12 out of 16 antigen/antibody complexes. For complexes for which ZDOCK produced one or more hits, the 2000 predictions are rescored using *E*_86_ and $E_{86}^{\text {trained}}$.

**Table 2:** Best rank for (re)scoring with ZDOCK, *E*_86_, and $E_{86}^{\text {trained}}$ for complexes from the Dockground 2.0 benchmark

Complex	ZDOCK	*E*_86_	$E_{86}^{\text {trained}}$
Enzyme/inhibitor			
1ARO	-	-	-
1AVW	2 (56/-)	12 (76/-)	46 (48/-)
1BTH	366 (-/-)	218 (-/-)	106 (-/-)
1CHO	3 (3/86)	5 (5/59)	1 (1/21)
1GPQ	1271 (-/-)	438 (-/-)	109 (-/-)
1ID5	72 (-/-)	11 (-/-)	4 (-/-)
1KU6	10 (62/-)	19 (74/-))	14 (103/-)
1OFH	-	-	-
1PPF	12 (12/36)	12 (12/109)	1 (1/13)
1T6G	2 (2/579)	22 (22/311)	5 (6/121)
1TX6	539 (610/-)	28 (28/-)	87 (1090/-)
1UGH	1 (1/1098)	1 (1/104)	1 (1/44)
1XX9	279 (-/-)	17 (-/-)	8 (-/-)
2BKR	4 (4/-)	18 (24/-)	33 (33/-)
2D26	-	-	-
2FI4	335 (1287/1287)	69 (69/182)	48 (48/86)
2KAI	269 (737/-)	287 (287/-)	75 (75/-)
3SIC	1 (6/24)	1 (1/1)	1 (1/1)
$\varnothing $	211.3	77.2	35.9
Antigen/antibody			
1A2Y	5 (-/-)	21 (-/-)	3 (-/-)
1G6V	1344 (-/-)	1485 (-/-)	705 (-/-)
1G9M	1 (5/-)	4 (38/-)	3 (18/-)
2BNQ	-	-	-
1BZQ	13 (13/13)	22 (22/61)	7 (43/274)
1FBI	609 (-/-)	1113 (-/-)	1174 (-/-)
1FNS	729 (-/-)	1055 (-/-)	1906 (-/-)
1H0D	159 (-/-)	17 (-/-)	1 (-/-)
1JTP	13 (-/-)	1 (-/-)	2 (-/-)
1MQ8	16 (98/-)	1479 (1548/-)	565 (807/-)
1NBY	-	-	-
1NCB	-	-	-
1NSN	562 (-/-)	695 (-/-)	949 (-/-)
1PKQ	-	-	-
1SQ2	1 (16/-)	1 (6/-)	2 (8/-)
1Z3G	6 (6/-)	1273 (1273/-)	378 (378/-)
$\varnothing $	288.2	597.2	474.6
Other complexes			
1BUI	343 (-/-)	1332 (-/-)	573 (-/-)
1F6A	-	-	-
1FM9	1 (14/52)	2 (3/26)	2 (24/87)
1G20	11 (132/-)	15 (178/-)	12 (34/-)
1G4A	-	-	-
1G4U	-	-	-
1GHQ	-	-	-
1GLB	1021 (-/-)	1356 (-/-)	1352 (-/-)
1HXY	-	-	-
1JWM	-	-	-
1K90	-	-	-
1K93	-	-	-
1L9B	-	-	-
1MA9	1 (4/-)	1 (3/-)	1 (1/-)
1NBF	91 (-/-)	105 (-/-)	106 (-/-)
1NVU	1020 (-/-)	555 (-/-)	192 (-/-)
1OMW	-	-	-
1OOK	171 (-/-)	639 (-/-)	237 (-/-)
1P7Q	3 (433/-)	13 (139/-)	21 (215/-)
1R4M	9 (-/-)	201 (-/-)	27 (-/-)
1RQQ	-	-	-
1S6V	1 (-/-)	9 (-/-)	7 (-/-)
1SQ0	-	-	-
1U0N	-	-	-
1U7F	149 (936/-)	1508 (1875/-)	969 (1740/-)
1UEX	25 (872/-)	14 (578/-)	11 (457/-)
1V7P	76 (-/-)	52 (-/-)	13 (-/-)
1WLI	-	-	-
1YI5	-	-	-
1ZY8	202 (325/435)	143 (527/1097)	54 (495/940)
2A42	-	-	-
2ATQ	-	-	-
2B4S	-	-	-
2CKH	1 (1/21)	1 (1/178)	1 (1/68)
2G45	912 (-/-)	682 (-/-)	438 (-/-)
2GD4	-	-	-
2GOO	-	-	-
2GY7	-	-	-
3FAP	143 (-/-)	80 (-/-)	95 (-/-)
3PRO	158 (350/-)	41 (154/-)	136 (412/-)
$\varnothing $	228.3	355.2	223.5

[a] The rank is set to 2,000 for calculating the average

$\varnothing $ indicates the average rank for the complex class in question. Targets without a hit in the top 2,000 are indicated by ‘–’. Values in brackets show the best rank for predictions with an IRMSD <2 Å and <1 Å, respectively. If such predictions are not found, no value is being reported

#### Enzyme/inhibitor complexes

**Fig. 7:**
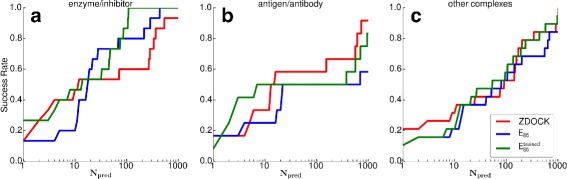
The success rate for the different complex complex classes, (**a**) enzyme/inhibitor, (**b**) antigen/antibody, and (**c**) other complexes for the Dockground test set when using ZDOCK (*red*), *E*_86_ (*blue*), and $E_{86}^{\text {trained}}$ (*green*) as (re)scoring function

Both OPEP-based rescoring functions can significantly improve the average ranking compared to ZDOCK, and $E_{86}^{\text {trained}}$ performs better than *E*_86_. Compared to ZDOCK, $E_{86}^{\text {trained}}$ can improve or maintain the rank for 11 targets and worsens the rank for 4 targets. However, for 1T6G the ranking decreased by only three places, from two to five. The standard soft rescoring function *E*_86_ can improve or maintain the ranking for 10 and worsens the ranking for 5 complexes. Figure 7a shows the success rate for the enzyme/inhibitor complexes. Both OPEP-based rescoring functions produce at least one hit in the top 1000 for all targets (i.e., the success rate is one for *N*_pred_=1000), which is not the case with ZDOCK. The performance of *E*_86_ is weak for *N*_pred_<10, but when considering more than 10 predictions the results improve, and *E*_86_ performs then better than ZDOCK and similar to $E_{86}^{\text {trained}}$. This means the selectivity of the *E*_86_ function near the native complex structure is not high enough; this is improved by training the scoring function, yielding $E_{86}^{\text {trained}}$. For *N*_pred_<10, the performance of $E_{86}^{\text {trained}}$ is equal or better to ZDOCK. This finding shows that training the OPEP-based scoring function was successful for the enzyme/inhibitor complex class.

#### Antigen/antibody complexes

ZDOCK finds hits in the top 2000 predictions for 12 out of 16 targets. $E_{86}^{\text {trained}}$ can improve or maintain the rank for five complexes. For 1G9M and 1SQ2, the rank only decreases from 1 to 3 and from 1 to 2, respectively. *E*_86_ performs less well, only improving the ranks of three targets and worsening them for the other nine targets. Figure 7b shows *E*_86_ has as many top-1 hits as ZDOCK has, but its success rate dwindles when more predictions are taken into account. $E_{86}^{\text {trained}}$, on the other hand, performs best when between 2 and 12 predictions are considered, yet for *N*_pred_>12 ZDOCK is still most successful for antigen/antibody complexes. Nonetheless, training *E*_86_ was worthwhile, as for *N*_pred_>1 the trained potential always performs better than or equal to *E*_86_, improving the average rank by more than 120 places (see Table 2).

#### Other complexes

For the other complexes, $E_{86}^{\text {trained}}$ can (considerably) improve the average ranking compared to ZDOCK and *E*_86_. Both $E_{86}^{\text {trained}}$ and *E*_86_ improve the ranks of nine targets and worsen them for the other 10. However, with *E*_86_ the ranking of these 10 targets is considerably increased, leading to an average rank more than 120 places higher than the ZDOCK average. Figure 7c shows that $E_{86}^{\text {trained}}$ performs slightly better than ZDOCK for *N*_pred_>20. However, the selectivity of $E_{86}^{\text {trained}}$ should be further improved for near-native predictions, i.e., its performance should be increased for the top 20 predictions. However, this may prove difficult, as the other complexes are a collection of protein–protein complexes from different classes. Thus, the protein–protein binding may be driven by different interactions for the different complexes, making it difficult to fully accommodate all peculiarities within a scoring function.

### Medium and high accuracy predictions

In the CAPRI evaluation [41], where the predictions are made blindly (i.e., without any knowledge of the correct answer), the predicted models are classified into four categories: incorrect, acceptable, medium, and high accuracy. To this end, the combination of three parameters is used, namely the fraction of native residue-residue contacts (*f*_nat_), the RMSD of the ligand molecules in the predicted versus the target complexes (LRMSD), and the IRMSD. A detailed description of these parameters and the corresponding thresholds used in classifying predictions can be found in previous CAPRI reports [41, 46]. In this work, only the IRMSD is used to assess the quality of the predictions. Application of *f*_nat_ requires an atomisitic representation of the predicted complexes as it is defined based on contacts between any atoms of interacting residues. Therefore, a transformation from the coarse-grained OPEP to an atomistic representation would be first required for the calculation of *f*_nat_. This would probably entail an optimization of the side chain positions so that the correct residue-residue contacts can form. While desirable, this is, however, would be beyond the scope of current study, which focuses on testing of OPEP as rescoring function for protein-protein docking. Therefore, only the IRMSD is used to classify the accuracy of predictions as high if IRMSD≤1 Å, medium if IRMSD≤2 Å, and acceptable if IRMSD<4 Å [41, 46]. As we want to know whether $E_{86}^{\text {trained}}$ finds more predictions of medium and high accuracy than *E*_86_, we determined the best ranks using these thresholds for the predictions obtained for the Dockground 2.0 test set. The results are listed in Table 2, together with the ones discussed above for threshold IRMSD<4 Å.

Table 2 reveals that one problem of our current approach is that ZDOCK does not produce many decoys of medium or high accuracy in the top 2000 predictions. This is particularly the case for antigen/antibody and other complexes. For only one antigen/antibody complex (1BZQ) decoys of high accuracy are predicted by ZDOCK, while for 5 of the 16 complexes predictions of medium accuracy are produced. For other complexes ZDOCK performs even worse as for only 9 (3) of the 40 complexes predictions of medium (high) accuracy are found. The ZDOCK results are somewhat better for the 18 enzyme/inhibitor complexes with decoys of medium accuracy being found for 10 complexes and of high accuracy for 6 complexes. As in the current study only energy minimization is used to optimize the geometry of the decoys, which has only minor effects on the docking pose (IRMSD changes of around 0.1 Å only, see Fig. 4), *E*_86_ and $E_{86}^{\text {trained}}$ cannot find more decoys of high or medium quality as being produced by ZDOCK. More structural refinement of the ZDOCK predictions, for instance by using MC simulations as done by RosettaDock [6, 47], would be necessary for their further improvement. Comparison of the ZDOCK, *E*_86_ and $E_{86}^{\text {trained}}$ scoring of decoys of medium accuracy and with top-10 ranks shows that $E_{86}^{\text {trained}}$ performs best for enzyme/inhibitor complexes. In this category, $E_{86}^{\text {trained}}$ ranks the docking models for four complexes first and for a fifth complex on sixth place. Also ZDOCK has for five complexes such models ranked in the top 10, however, for none on the first place. *E*_86_ predicts for only three complexes top-10 ranks, however, for two of them they are on the first place. For antigen/antibody complexes, ZDOCK finds for two complexes models in the top-10 rank list, while *E*_86_ and $E_{86}^{\text {trained}}$ for only one complex. For other complexes, *E*_86_ is slightly better than ZDOCK and $E_{86}^{\text {trained}}$ as it has for three complexes decoys in the top-10 list, while the other two scoring functions achieve this for only two complexes.

In summary, *E*_86_ and $E_{86}^{\text {trained}}$ rank docking models of medium accuracy on average better than ZDOCK (apart from antigen/antibody complexes). For complex 3SIC both OPEP-based rescoring functions even rank a high-accuracy decoy first, which ZDOCK fails to achieve for any complex; it does not even place any decoy of high accuracy in the top 10. However, mainly due to limited refinement of the docking models obtained from ZDOCK, both *E*_86_ and $E_{86}^{\text {trained}}$ do not find quantitatively more docking models of medium and high quality. This is further supported by the fact that for seven complexes (1BTH, 1XX9, 2BKR, 1G20, 1NBF, 1OOK, 3PRO), for which no decoy of high (sometimes not even of medium) accuracy and also no top-10 hit are found after rescoring, the native (i.e., target) complex is ranked first or second by *E*_86_ and/or $E_{86}^{\text {trained}}$ (data not shown). In these cases rescoring with *E*_86_ and $E_{86}^{\text {trained}}$ would have worked if the correct decoys had been generated. It should be noted, however, that in many other cases the native complex has a much higher rank than the other decoys, also for complexes for which top-10 predictions of medium or even high accuracy have been found. A similar observation was made by Baker and co-workers [6] when the performance of RosettaDock was for the first time tested. There, the problem was solved by performing 50 rounds of side-chain repacking and minimization. We assume that also after the transformation of the PDB structures to the coarse grained representation, energy minimization is often not sufficient for an optimal positioning of the side-chain beads. In our future work we will test wether side-chain refinement will improve the scoring of the native complexes.

## Discussion and conclusion

In this work we examined the applicability of the coarse-grained OPEP force field [22] for refining and rescoring rigid body protein–protein docking predictions. We use ZDOCK [11] to produce protein complex predictions, which also serves as quality control. The predictions from the ZDOCK benchmark 4.0 are transformed to the coarse-grained model and their energy minimized using the original OPEP potential, which is followed by rescoring with a softer energy function, denoted *E*_86_, based on the interprotein OPEP interactions. This approach produces a better rank for the best prediction than ZDOCK for 54 % of targets. However, the results differ significantly across the three complex classes: There is an improvement for 65 % of the enzyme/inhibitor complexes, for 55 % of other complexes, but for only 32 % of the antigen/antibody complexes. Furthermore, the average rank with *E*_86_ is for antigen/antibody complexes considerably higher than that obtained with ZDOCK. To improve these results, we developed a training scheme for the OPEP-based rescoring function based on false positive and false negative predictions. The resulting trained rescoring function callesd $E_{86}^{\text {trained}}$, which was applied to the targets from the test dataset taken from the Dockground benchmark [24], produces a lower best rank compared to the ZDOCK results for 54 % of the targets, while the untrained OPEP-based scoring function can only improve the rank of 48 % of targets. The trained scoring function performs particularly well for enzyme/inhibitor complexes, where the best rank of 73 % of targets can be improved. These figures are 47 % for other complexes, and 42 % for antigen/antibody complexes.

***Performance analysis for different complex classes*** Training the OPEP-based rescoring function revealed that the complexes from different classes are stabilized by different protein–protein interactions. For enzyme/inhibitor and other complexes, interactions between hydrophobic residues are of general importance, and for enzyme/inhibitor complexes backbone–backbone hydrogen bonds are also important. For antigen/antibody complexes we found that training strengthens the interactions between residues, which have been previoulsy shown to be prevalent at the paratope of the antibody and the epitope of the antigen [42, 45]. The different performance and training potentials reflect the different protein–protein binding in enzyme/inhibitor and antigen/antibody complexes. Antibodies can recognize a wide spectrum of antigens, including proteins, polysaccharides, nucleic acids, and even lipids, while enzyme–ligand binding has developed in an evolutionary sense to enable specific binding of a ligand to its target enzyme. This diverse binding by antibodies is accommodated by the complementarity determining regions composed of six loops that are modified in shape and chemical nature to match the corresponding features of the antigen epitope. Furthermore, the paratopes are mainly discontinuous, and binding is usually mediated by only 4–13 residues. In contrast, the enzyme inhibitors are typically small proteins that form tight, substrate-like interactions with the enzyme, which is reflected in a much stronger binding energetics. The binding constants for enzyme/inhibitor complexes are in the femtomolar range, which is about six orders of magnitude smaller than the nanomolar binding constants between antigen and antibody [42]. Thus, it is not surprising that the more static and strong enzyme–inhibitor binding is more easily predicted than the protein–protein interface in antigen/antibody complexes, where already one missing or one wrong interresidue contact in a decoy can have a profound impact on the performance of the scoring result. Our results suggest that the collective complex class called ‘other complexes’ lies between the two ends of the spectrum bounded by enzyme/inhibitor and antigen/antibody complexes.

***Comparison to other rescoring approaches*** In summary, this study demonstrates for the first time that energy functions derived from the coarse-grained OPEP force field can be employed to rescore predictions for protein–protein complexes. This expands the applicability of OPEP to new problems. While the performance of OPEP is already very good for enzyme/inhibitor complexes and better than ZDOCK, for the other complexes and especially for antigen/antibody complexes, ZDOCK is still better suited. The comparison to RDOCK results [13] shows that rescoring with an all-atom force field works somewhat better than rescoring with *E*_86_ and $E_{86}^{\text {trained}}$. In RDOCK, the ZDOCK predictions are subjected to a three-stage energy minimization scheme using the CHARMM force field [48] and amounting to 130 minimization steps, followed by the rescoring based on the CHARMM electrostatic and desolvation energies. This elaborate approach improves the success rate for *N*_pred_=10 (i.e., the success rate for finding a near-native hit within the first 10 predictions, as expected in the CAPRI experiment [41]) from 38 to 45 % for decoys obtained from ZDOCK(PDE), which is similar to the ZDOCK 3.02 version used in this work. In our study, the success rate for *N*_pred_=10 decreases by 1–2 % after rescoring with *E*_86_ and $E_{86}^{\text {trained}}$ (see Figs. 3 and 7). In case of *E*_86_ it is due to the poor performance of this scoring function for antigen/antibody complexes, while $E_{86}^{\text {trained}}$ does not perform well for *N*_pred_<12 for other complexes. It should be noted that also RDOCK is considerably less successful for antigen/antibody complexes compared to enzyme/inhibitor complexes, supporting our conclusion that the rescoring of the former is more challenging than that of the latter.

Comparison to other coarse-grained force fields shows that OPEP is better suited as scoring function for protein-protein docking than these. In addition to OPEP we also tested the coarse-grained force field developed by Bereau and Deserno (BD) [49] on a decoy set produced by ZDOCK consisting of 23 enzyme/inhibitor and 23 other complexes. The BD force field increased the rank of 31 complexes and decreased it for only four complexes, which is considerably worse than what we obtain with OPEP. Reasons for the failure of the BD force field when applied to protein-protein docking are that the side-chain beads have all the same size and that electrostatic interactions between charged residues are not modelled, features that are present in OPEP. Moreover, in a study performed similarly to ours, the UNRES coarse-grained force field was tested as rescoring function [50]. The number of hits that were retained in the top-10 predictions generated by FTDock [51] decreased by more than 50 %, while with our approach the success rate decreases by only 1–2 % at *N*_pred_=10. This shows that while OPEP is still not a perfect scoring function for protein-protein docking, it is clearly better suited than other coarse-grained force fields.

***Outlook*** In our future work we strive to further improve the performance of the OPEP rescoring functions. Here, special attention will be devoted to antigen/antibody complexes, where improvement is most needed. In addition, we will not only rescore the decoys generated by ZDOCK but also refine them by performing Monte Carlo simulations with OPEP. One advantage of OPEP is that it is a physics-motivated force field defined based on continuous functions and is therefore ideally suited for flexible docking. Our aim is to produce a reliable refinement and rescoring protocol based on OPEP that only needs docking decoys generated by ZDOCK or another global search algorithm as additional input. For the participation in the CAPRI experiment, however, a final transformation from the coarse-grained to the atomistic level for the top-10 decoys will become necessary as only atomistic decoys can be submitted.

## Availability of data and materials

All decoy structures as obtained from ZDOCK and after their transformation to the OPEP coarse-grained level are available upon request from the authors.
